# Editorial: Allergies and anti-allergy drug discovery in Africa

**DOI:** 10.3389/falgy.2025.1593023

**Published:** 2025-04-04

**Authors:** Sabelo Hadebe, Andrew Glory Mtewa, Mushagalusa Kasali Félicien, Jonathan Peter, Precious Takondwa Makondi

**Affiliations:** ^1^ Division of Immunology, Department of Pathology, Faculty of Health Sciences, University of Cape Town, Cape Town, South Africa; ^2^ Chemistry Section, Institute of Technology, Malawi University of Science and Technology, Limbe, Malawi; ^3^ The Drug Discovery Group, Malawi University of Science and Technology, Limbe, Malawi; ^4^ Department of Pharmacy, Official University of Bukavu, Bukavu, Democratic Republic of Congo; ^5^ Division of Allergology and Immunology, Department of Medicine, Faculty of Health Sciences, University of Cape Town & Lung Institute, University of Cape Town, Cape Town, South Africa; ^6^ National Cancer Center, Kamuzu Central Hospital, Lilongwe, Malawi

**Keywords:** ligand based drug design, medicinal chemistry studies, development of datasets, pharmacology of allergies, drug reaction - allergy

**Editorial on the Research Topic**
Allergies and anti-allergy drug discovery in Africa

Allergic diseases are on the rise in Africa, coinciding with population growth in urban or semi-urban industrialized cities. The risk factors that predispose African populations to allergic diseases are poorly described. We felt that there was a need to compile research and review articles that address allergy in Africa to help fill this information gap.

There is a paucity of data on the immunogenetics and pharmacogenetics of asthma in people of African descent, which has implications for drug dosing and tailored therapeutic doses. Mabelane et al. reviewed the literature on the prevalence of asthma in Africa and the diaspora. While access to and adherence to asthma drugs may affect treatment outcomes, there is a reasonable number of patients who do not respond to these medications. Minor allele frequency differences at pharmacogenetic loci between populations may explain treatment failure or adverse reactions in people of African descent or admixed populations. There are several minor allele frequencies that have been identified in the inhaled corticosteroid, short-acting β-adrenergic agonist (SABA) and long-acting β-adrenergic agonist (LABA) pathways. These variants are associated with either reduced salmeterol/formoterol (in the case of LABA), albuterol (in the case of SABA) or corticosteroids. The study highlighted key variants of African descent in each of these pathways that may influence lung function and worsen asthma and the possibility of these variants being used as predictive markers of corticosteroid response.

Lunjani et al., discussed phenotypes, endotypes and genotypes of atopic dermatitis (AD) in African populations. One of the challenging aspects of diagnosing AD in African populations is the use of Hanifin and Rajka criteria for clinical diagnosis of AD. Although this method has been improved by the UK Working Party, it is still a poor predictor of AD in African populations. Both the Eczema Area and Severity Index (EASI) and SCORing Atopic Dermatitis (SCORAD) tests rely on indices or outcomes such as erythema which are not readily assessed in individuals with dark skin pigmentation. This inevitably leads to a gross underestimation of AD and its severity in African populations. This further limits personalized treatment strategies and exacerbates health care disparities in African populations compared to their European population counterparts.

AD is morphologically and phenotypically different in African populations. It is mainly characterized by the incomplete maturation of keratinocytes and an atypical morphology similar to that of psoriasis. Other morphological features include densely aggregated follicular papules, scaling, treatment-resistant lichenification, pigmentary changes, and higher levels of transepidermal water loss. Other notable differences in African populations compared to European populations are filaggrin gene mutations and AD prevalence. In African populations Filaggrin gene mutations are less associated with AD risk. It is possible that other polymorphisms at other loci within the filaggrin gene complex may explain AD risk in African populations, but the lack of larger genome-wide association studies hinders progress. There is a dearth of information regarding AD immune profiles in African populations. Children in African populations present with higher IgE and greater sensitization to food allergens, which may correlate with polymorphisms in IL-4 and IL-10 in these children. In both adult and childhood AD, African populations show a stronger presence of T helper (TH) 2 accompanied by TH17 and TH22 cells. These differences may be attributable to geographical location, dietary intake, and large differences in microbiota in these populations, which influence both gut and skin commensals and their metabolites that interact with immune cells. The study highlights the need to study the collective influence of local and specific environmental exposures, genetics, epigenetics, immune responses and socio-economic status on AD prevalence in African populations.

Isaacs and Lehloenya discussed HIV-associated photodermatitis in African populations. HIV photodermatitis is a complex group of skin conditions caused by an abnormal or excessive reaction to sunlight. In people living with HIV/AIDS (PLWHA), photosensitivity is 7 times more common in pigmented skin. Photodermatitis can be classified into 5 types, immunologically mediated, drug and chemical-induced, photo-aggravated, photosensitivity associated with defective DNA repair mechanisms and metabolic photodermatitis. The authors further defined a spectrum of clinical features of HIV photodermatitis that included Chronic actinic dermatitis, Actinic lichenoid leukomelanoderma, HIV photodermatitis with depigmentation, Actinic lichen planus, Porphyria cutanea tarda and Pellagra. The authors highlighted the difficulties in diagnosing individuals with pigmented skin, which are further worsened by its underrepresentation in textbooks and databases. Diagnostic challenges include difficulty in detecting erythema and purpura in melanin-rich skin, susceptibility to dyspigmentation in darker-skinned individuals, contrast between normal and dyspigmented skin that may negatively affect patients with darker skin, higher than normal pruritus in darker-skinned individuals and greater mast cell degranulation patterns associated with higher TH2 cell proliferation and IgE in PLWHA. The authors argue that although there is a significant clinical overlap between HIV photodermatitis conditions, a good clinical history, clinical examination with the assistance of skin biopsy, and photo testing are helpful in identifying photodermatitis and in distinguishing phototoxic from photoallergic reactions.

Konyana et al., in their research article investigated cutaneous immune-mediated adverse drug reactions (CADRs) in PLWHA who had been admitted to the Nelson Mandela Academic Hospital due to Severe cutaneous adverse drug reactions (SCARs). In this retrospective and prospective study, the authors recruited 122 patients with CADRs and evaluated the most common offending drugs. The most common SCAR phenotype was Stevens-Johnson syndrome/toxic epidermal necrolysis (SJS/TEN) in 59.8% of the patients (73/122). The most common offending drug was cotrimoxazole in 24.6% of the patients (30/122) followed by anti-retroviral therapy (ART) in 13.9% (17/122). The offending drug was not identified in almost a quarter of the cases, which was a limitation of the retrospective study. The study highlights the need for timely prospective identification of SCAR and CADR cases to reduce mortality and morbidity in vulnerable PLWHA.

In this editorial, we have highlighted the work done noting the rising prevalence of allergic diseases in Africa and emphasized the need for more research to address the existing information gap on immunogenetics, pharmacogenetics, and tailored treatments for African populations. We have noted findings that include differences in asthma drug response due to genetic variants, challenges in diagnosing atopic dermatitis (AD) in dark-skinned individuals, and the complexity of HIV-associated photodermatitis. We have also underscored the importance of studying genetic, local environmental and socioeconomic factors that influence allergies. Based on the work done, we recommend further genome-wide association studies, improved diagnostic criteria for AD, and timely identification of adverse drug reactions in HIV patients to reduce morbidity and mortality.

